# Endocrown Feasibility for Primary Molars: A Finite Element Study

**DOI:** 10.1055/s-0043-1764421

**Published:** 2023-05-02

**Authors:** Aeshah Hassan Abduljabar, Ahmad Waleed Iskander, Mohamed Taha Elfezary, Mohamed AboElkasem Ahmed Wakwak, Wafa Abdullah Bathabt, Yasser R. Souror

**Affiliations:** 1 Batterjee Medical College, Jeddah, Saudi Arabia; 2 Pediatric Dentistry Department, Faculty of Dentistry, Al-Azhar University, Assiut, Egypt; 3 Misr University for Science & Technology (MUST) 6th of October City, Giza, Egypt

**Keywords:** primary molar, endocrown, zirconia, E-max, finite analysis

## Abstract

**Objective** To study the possibility of using pediatric endocrowns to restore the second primary molar using three-dimensional (3D) finite element analysis.

**Design** A 3D finite element model was built for a pediatric mandibular molar, starting with laser scanning a naturally extracted tooth. The access cavity had an elliptic shape with 6 mm width, 4 mm height, and 2 mm depth with a wall taper angle of 5 degrees.

Two materials (Zr and E-max) were tested for the endocrown and two cementing materials (glass ionomer and resin cement) with 20 to 40 μm thickness. Twelve case studies were reported within this research as the applied load of 330 N was tested with three angulations vertical, oblique at 45 degrees, and laterally.

**Results** Twelve linear static stress analyses were performed. The resultant stresses and deformations' distribution patterns did not alter much, and values were within the threshold of physiological tolerance. Deformations were negligibly changed with changing endocrown and cement materials. In contrast, endocrown stresses indicated zirconia endocrown would have a long lifetime, while E-max one will have a relatively short lifetime.

**Conclusions** Analysis results indicated that bone was negligibly affected by changing endocrowns and cementing materials. Both tested endocrown materials can be used safely. Zirconia endocrowns may have a much longer lifetime than E-max.

## Introduction

Dental caries in young children may adversely affect primary molars, which are highly important in mastication, nutrition, development of normal occlusion, development of a normal healthy child, and quality of life. 1 So, every effort should be made to preserve them. Caries progression in primary teeth is rapid due to thinner, more porous, and less mineralized enamel. 2 This can result in bacterial spread to the pulp causing dental infection (cellulitis or abscess) if not treated.

Primary teeth with small caries lesions could be treated with caries excavation, cavity preparation, and cavity restoration using materials such as composite resins or glass ionomers. 3 Therefore, a proper caries excavation followed by appropriate restoration is substantial. 4

For large caries lesions in primary molars, various options are available such as full-coverage restorations, which include stainless steel crowns (SSCs), pre-veneered SSCs, and zirconia crowns. SSCs have been the most widely used coverage crowns for the restoration of primary molars for over 50 years. 5 Numerous studies concluded the success of SSCs in restoring decayed primary molars. Despite all these advantages, SSCs have significant drawbacks; their poor esthetic appearance and relative application difficulty. 6 Nowadays, patients and guardians demand more esthetically appealing restorations. 7

Regarding prefabricated zirconia crowns, they offer an excellent aesthetic alternative to SSC. However, they cannot be crimped, contoured, and passively fit. 8 Moreover, the application of zirconia crowns in most cases requires more tooth structure removal and thus it is associated with a higher number of complications than SCC including higher enamel wear of opposing teeth and higher cost. The high cost also limits the use of zirconia crowns for restoring primary molars with large caries lesions. 9

An alternative to overcome the disadvantages of the zirconia material is the endocrown. Pissis described endocrowns in 1999 as “adhesive endodontic crowns.” They are anchored to the pulp chamber and cavity margins so that macromechanical retention is provided by friction between the surface of the crown and the pulpal walls, and the adhesive cement provides micromechanical retention. 10 Endocrowns are less expensive, require less tooth preparation time and is easier to apply and provide good esthetics. 11

Results from a previous study showed that endocrowns used to restore endodontically treated permanent teeth can be applied to primary teeth, especially in cases with excessive loss of coronal dental tissue and limited interocclusal space. 7

Finite element analysis (FEA) has been broadly utilized through numerical analysis and effectively applied in many engineering and bioengineering areas since the 1960s. It has been involved in many studies in dentistry that can assist the mechanical engineering principles present in these specialties, preferring the use of finite element modeling. It includes a series of computational procedures to measure the stress in every component, which plays out a model arrangement. Such an analysis permits the assurance of stress coming from the external force, pressure, thermal change, and other elements. 12

In pediatric dentistry, ceramic endocrown restorations are considered an alternative to prefabricated zirconia crowns. However, only a few studies have been conducted to assess the use of endocrowns in the primary dentition. So, this study was conducted to compare equivalent stresses in second primary molars restored with two different material endocrowns during masticatory simulation using FEA.

## Materials and Methods

A 3D finite element model for the primary second mandibular molar was developed by laser scanning for freshly extracted sound tooth due to periodontal disease after parent acceptance.

The tooth geometry was acquired using a laser scanner (Geomagic Capture, 3D Systems, Cary, NC, USA). Such a scanner produced a data file containing a cloud of points coordinates. An intermediate software (Rhino 3.0-McNeel inc., Seattle, WA, USA) was required to trim the newly created surfaces by the acquired points. The tooth geometry was transferred to ANSYS (finite element package) as a STEP file format. 13

In contrast, cortical and cancellous bone models were created by commercial computer-aided design software Autodesk Inventor software version 8.0 (Autodesk Inc., San Rafael, CA, USA). The bone geometry was simplified and simulated as two coaxial cylinders. The inner one represents the cancellous bone with a 14 mm diameter and 22 mm height that fills the internal cylindrical space of the other cylinder (shell of 1 mm thickness) that represents cortical bone (outer diameter of 16 mm and height of 24 mm). 14 15

A cement layer of 20 to 40 μm thickness 16 17 was placed under the endocrown. Creating such geometry was done via a set of Boolean operations on the ANSYS environment (ANSYS Inc., Canonsburg, PA, USA) to finalize the model. Endocrown has a 2 mm thickness, while the access cavity has an elliptic shape of 6 mm width, 4 mm height, and 2 mm depth with a wall taper angle of 5 degrees. All materials were considered isotropic, linear, and elastic, fed to ANSYS as listed in Table 1 .

**Table 1:** Material properties used in the finite element model(s) 18

Material	Young's modulus [GPa]	Poisson's ratio
**Crown:** Zirconia (Zr)	210	0.30
E-max	70	0.30
**Cement:** Glass ionomer	12.0	0.25
Resin cement	7.0	0.27
Dentine	18.6	0.31
Enamel	84.1	0.30
Cortical bone	13.7	0.30
Cancellous bone	1.37	0.30

**Fig. 1:**
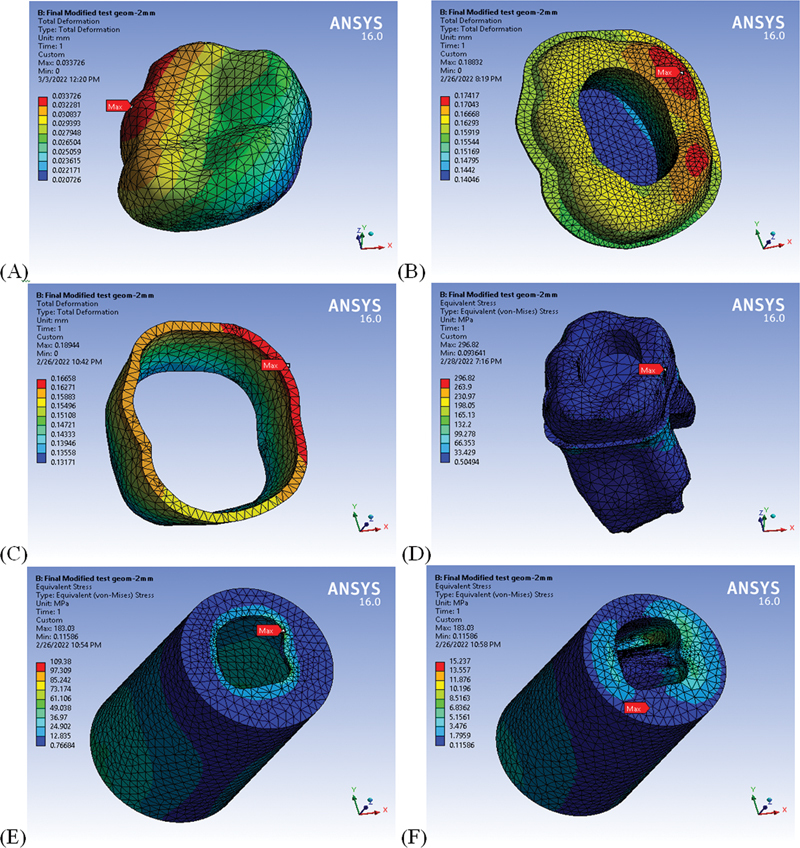
Screenshots from runs for model components; total deformation ( **A** ) endocrown, ( **B** ) cement layer, ( **C** ) enamel and **Von Mises stress,** ( **D** ) Dentine, ( **E** ) cancellous bone, ( **F** ) cortical bone.

The meshing of the model's components was done by 3D brick solid element “187,” which has three degrees of freedom (translation in main axes directions). 15 The numbers of elements and nodes of each structure are listed in Table 2 , and the meshed model components are presented as screenshots from ANSYS in Fig. 1 .

**Table 2:** Mesh density of components of two models

	Model 1	
Material	Number of elements	Number of nodes
Endocrown	48,116	33,615
Cement (20–40 μm)	23,485	11,699
Dentine	12,817	7,442
Enamel	113,942	80,506
Cortical bone	21,956	11,315
Cancellous bone	241,570	175,843

**Fig. 2:**
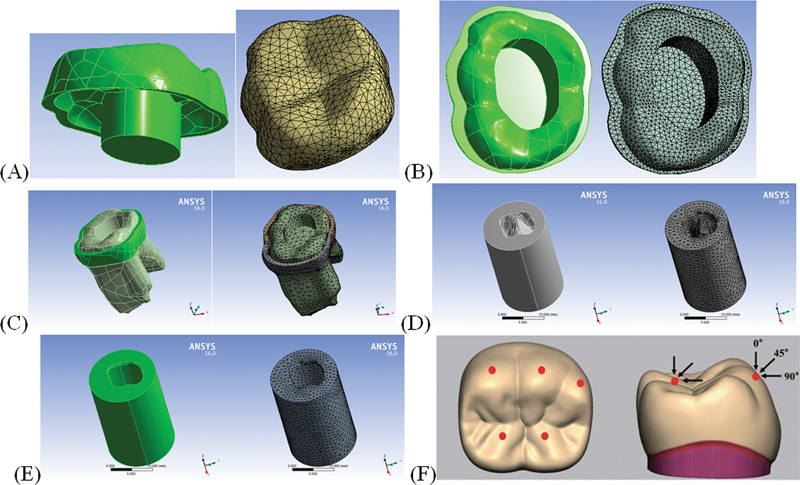
Mesh density of different components of the model. ( **A** ) Endocrown, ( **B** ) cement layer, ( **C** ) tooth structure, ( **D** ) cancellous bone, ( **E** ) cortical bone, ( **F** ) loading points. 19

Three loading cases were studied for each endocrown and cement material combination; the load of 330 N was applied vertically, obliquely, and laterally. Three points on the outer inclines of the buccal cusps and two points on the inner inclines of the lingual cusps were loaded ( Fig. 2F ). The model was verified by comparing it with the model described in similar studies 15 18 19 before extracting analysis results.

The boundary condition was defined by setting the lowest plane in each model to be fixed in place. A personal computer (Intel Core i7 processor, 2.4 GHz, 6.0 GB RAM) was utilized for performing the linear static analyses on ANSYS.

## Results

**Fig. 3:**
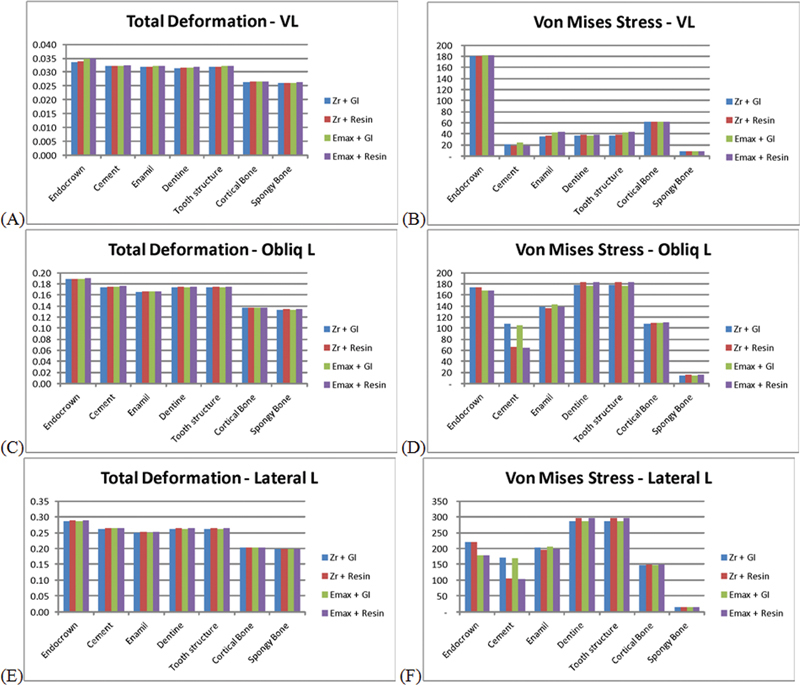
Comparison of different materials stresses under different angles loading. **Vertical loading** ( **A** ) total deformation, ( **B** ) Von Mises stress; **oblique loading** ( **C** ) total deformation, ( **D** ) Von Mises stress; and **lateral loading** ( **E** ) total deformation, ( **F** ) Von Mises stress.

Twelve linear static analyses were performed to evaluate the total deformation and Von Mises stress and compare their values to extract conclusions. Figure 1 demonstrates these result distributions on all parts of the model. Figure 3 compared the extreme values of total deformation and Von Mises stress obtained in the 12 cases (runs). Changing endocrown material and/or cement material did not change deformations or stress distributions but altered its values.

Total deformation under the exact loading condition comparisons ( Fig. 3A, C, E ) showed that changing endocrown and cement materials did not change much (about 2 microns of variation). Vertical loading resulted in the lowest deformation, followed by oblique one, while lateral loading resulted in the highest deformation.

Whatever the loading condition, the total deformation decreases from endocrown, then cement, followed by dentine and enamel (or tooth structure). Cortical and cancellous bones were insensitive to these material changes.

Von Mises stresses comparison of endocrown and cementation materials showed little difference under vertical loading. Increasing loading angulation to oblique and then lateral increased the difference between the two endocrown materials, in which E-max received fewer stresses. In contrast, the E-max stress level was close to yield stress, which indicated a short lifetime. Glass ionomer received more stress than the resin cement under the three tested loads, where the difference increased with increasing loading angulation.

Enamel is slightly affected by changing endocrown and cement materials but increasing the applied load angulation generally increases the stresses received by enamel. Dentine reaches critical stress levels under lateral loading, regardless of the combination of endocrown and cement materials. The enamel Von Mises stress under vertical load was of order 40 MPa, which jumped to 140 MPa under oblique loading and then to 200 MPa under lateral loading.

Bone generally was not sensitive to changing endocrown and cement materials. Cortical bone Von Mises stress was within physiological limits under vertical and oblique loading cases. Still, it tended to be closer to the yield point (120–150 MPa), i.e., it exceeded the fatigue limit = 75–100 MPa, with increasing load angulation.

## Discussion

Although SSC is the most common restoration of endodontically treated primary molars, there is a need for an esthetic restoration. Endocrown is an esthetic partial coverage restoration with an acceptable success rate for endodontically treated permanent molars. 20 21

This study was conducted to explore the stress distribution on primary molars restored by endocrowns made from Zirconia and E-max using FEA.

Regarding stress analysis, Von Mises stress analysis was conducted in this study as it is an indicator of the probable deterioration occurrence, and the maximum principal stress is accepted as an adequate index to determine the failure of brittle materials. In our study, Von Mises stresses comparison of endocrown and cementation materials showed little difference under vertical loading.

In this study, we compared E-max and zirconia endocrown designs with different cement materials (GIC and RC). The results showed that changing the endocrown material and/or cement material did not change deformations or stress distributions, which indicates similar outcomes of all tested materials with different types of cement. 22

However, there is a slight change in deformation values (about 2 microns of variation) under the same loading condition. This may be explained on the basis that the masticatory force applied was relatively small to represent the masticatory force of children. This low magnitude of load may have an insufficient impact to induce a difference in deformations or stress distributions between the tested materials. In addition, the similarity in properties of both E-max and zirconia may explain the insignificant difference between deformation and stress distribution. In contrast, the results of this study showed that the E-max stress level was close to yield stress, which indicates a short lifetime of E-max over zirconia. 23

Regarding the direction of load outcomes, the present study showed that increasing applied load angulation increases the stresses received by tooth structures and bone. This means that the lateral load is more destructive, which may be due to the non-axial force subjecting the tooth structure to torquing and offloading. 24 25

Regarding the cement materials used, the results of the current study showed that GIC was found to receive about 60% more stress than resin cement. This may be due to the modulus of elasticity of GIC being higher than that of RC. 26 However, both types of cement have a reasonable lifetime before failure. 19 27

The current study showed that the primary molar tooth structure could support the endocrown under the specified load. Considering that under lateral loading, dentine reached critical stress levels. As a result of masticatory forces in the lateral direction, which are concentrated in a smaller area, the dentine underwent the most stress. Consequently, adequate tooth structure should be left at the cuspal inclines most susceptible to lateral forces to withstand the resulting stress. Evidence suggests that a crown is more likely to endure masticatory forces when restoring a tooth. 28

This study showed that bone was not sensitive to changing the endocrown and cement materials. These results agreed with the study of El-Anwar MI, who reported that bone was insensitive to the cement type. Locations of extreme stresses and deformation did not change at the crest of cortical bone. 16

The results of our study are in accordance with those reported by Dejak **.** Ceramic endocrowns in molars showed the lowest Von Mises stress and were less prone to fracture or debonding under physiological loads. 29

Other studies reported the possibility of restoring primary molars with ceramic endocrowns for short- to long-term treatment, taking into consideration the high fracture strength of ceramics, minimally invasive preparation, and no gingival trauma. 30 31 32

## Limitations of the Study

It was a preliminary force analysis study where the software was used without laboratory or clinical investigation. Furthermore, it was confined to a single cavity design to a single tooth (lower second primary molars). So, further investigations ( *in vivo* and *in vitro* ) are required for confirmation of the feasibility of using endocrowns in primary molars
